# Diagnosis and Prognosis of Sepsis Based on Use of Cytokines, Chemokines, and Growth Factors

**DOI:** 10.1155/2019/1089107

**Published:** 2019-09-08

**Authors:** Dong Wook Jekarl, Ji Yeon Kim, Jick Hwan Ha, Seungok Lee, Jaeeun Yoo, Myungshin Kim, Yonggoo Kim

**Affiliations:** ^1^Department of Laboratory Medicine, Seoul St. Mary's Hospital, College of Medicine, The Catholic University of Korea, Seoul 06591, Republic of Korea; ^2^Laboratory for Development and Evaluation Center, College of Medicine, The Catholic University of Korea, Seoul 06592, Republic of Korea; ^3^Department of Laboratory Medicine, Incheon St. Mary's Hospital, College of Medicine, The 23 Catholic University of Korea, Seoul 06591, Republic of Korea; ^4^Department of Internal Medicine, Incheon St. Mary's Hospital, College of Medicine, The Catholic University of Korea, Incheon 21431, Republic of Korea

## Abstract

The focus of sepsis has shifted from inflammation to organ dysfunction on the basis of a recent definition based on the sequential organ failure score (SOFA). A diagnostic and prognostic marker is necessary under this definition but is currently unknown. We enrolled 80 sepsis patients consecutively admitted to an intensive care unit through the emergency department and 80 healthy control patients who received routine health check-ups from August 2018 to January 2019. SEPSIS-3 criteria were used for the diagnosis of patients based on SOFA score ≥ 2 from the baseline along with evidence of infection. Concentrations of 28 cytokines, eight chemokines, and nine growth factors were measured on the day of diagnosis. Hierarchical cluster analysis was performed for molecules. The majority of infections were pneumonia (45% of patients) and urinary tract infections (40% of patients). Most of the measured molecules were increased in patients with sepsis. Area under receiver operating characteristic curve (AUROC) values were found to be as follows: hepatic growth factor (HGF), 0.899; interleukin-1 receptor antagonist (IL-1RA), 0.893; C-C motif ligand 5 (CCL5) 5, 0.887; C-X-C motif chemokine 10 (CXCL10), 0.851; CCL2, 0.840; and IL-6, 0.830. IL-1RA, IL-6, IL-8, IL-15, and CCL11 concentrations correlated with SOFA score with statistical significance. Prognosis multivariate analysis revealed an odds ratio of 0.968 for epidermal growth factor (EGF). Three clusters were formed, of which Clusters 2 and 3 were associated with nonsurvivors. Diagnosis of sepsis was performed using cytokines, chemokines, and growth factors. HGF revealed the highest diagnostic capability, and EGF predicted favorable prognosis among the tested molecules.

## 1. Introduction

Sepsis is an organ dysfunction caused by a dysregulated host response to infection [1, 2]. The global population incidence rates of sepsis and severe sepsis were 288 and 148 per 100,000 person years, respectively, from 1979 to 2015 and have increased to 437 and 270 per 100,100 person years, respectively, during the last decade [3]. Hospital mortality rates of 17% and 26% for sepsis and severe sepsis, respectively, were reported.

Immune dysregulation represents an imbalance of proinflammatory and anti-inflammatory aspects of immune function or of innate immunity- and adaptive immunity-related functions [4–7]. Proinflammatory cytokines include tumor necrosis factor-alpha (TNF-*α*), interleukin- (IL-) 1, IL-6, IL-12, IL-17, interferon-gamma (IFN-*γ*), and macrophage migration inhibitory factor. Anti-inflammatory cytokines include IL-4, IL-10, and transforming growth factor-beta (TGF-*β*) [5, 8]. In sepsis, a proinflammatory state followed by an anti-inflammatory state was implicated as one mechanism of pathogenesis. However, these states were not temporal and separate but were rather simultaneously overlapping [8, 9]. The anti-inflammatory state or immune suppression is associated with a decreased level of human leukocyte antigen-DR isotype, impaired cytokine production, and increased concentrations of IL-10, TNF receptors, IL-1 receptor antagonist (IL-1RA), and IL-1 receptor type II [9, 10].

Molecules associated with sepsis include chemokines and acute phase reactants. In sepsis patients, a subset of CC motif chemokines, including CCL1, CCL2, CCL8, CCL20, or CXC motif chemokines that include CXCL8, CXCL10, and CXCL12, along with cytokines is increased compared to normal controls [11]. Procalcitonin (PCT) and C-reactive protein (CRP) are acute phase proteins that have been evaluated for use in the diagnosis and prognosis of sepsis [12, 13] and have been included in the diagnostic criteria for sepsis by the Surviving Sepsis Campaign [7].

Various molecules, including cytokines, chemokines, and acute phase reactants are involved in these processes that are interactive and dynamic [8]. These molecules are utilized in diagnosis, prognosis, etiology, and response to therapy [14]. Recently, the definition of sepsis was changed from systematic inflammation with evidence of infection to life threatening organ dysfunction by dysregulation of host response with evidence of infection according to the SEPSIS-3 criteria [1, 15, 16]. Therefore, molecules involved in diagnosis and prognosis might have to be different from the previous literature.

In this study, we evaluated the levels of 45 molecules that are typically examined in sepsis patients, which included cytokines, chemokines, and growth factors. Their levels were also determined in healthy normal controls. Twenty-eight cytokines included interferon- (IFN-) *α*, IFN-*γ*, IL-1*β*, IL-1*α*, IL-1RA, IL-2, IL-4, IL-5, IL-6, IL-7, IL-8, IL-9, IL-10, IL-12, IL-13, IL-15, IL-17*α*, IL-18, IL-21, IL-22, IL-23, IL-27, IL-31, leukemia inhibitory factor (LIF), granulocyte macrophage colony stimulating factor (GM-CSF), stem cell factor (SCF), tumor necrosis factor- (TNF-) *α*, and TNF-*β*. Eight chemokines included CCL2, CCL3, CCL4, CCL5, CCL11, CXCL1, CXCL10, and CXCL12. Nine growth factors included brain-derived neurotrophic factor (BDNF), epidermal growth factor (EGF), fibroblast growth factor- (FGF-) 2, hepatocyte growth factor, nerve growth factor- (NGF-) *β*, platelet-derived growth factor- (PDGF-) BB, placental growth factor (PLGF), vascular endothelial growth factor- (VEGF-) A, and VEGF-D.

## 2. Materials and Methods

### 2.1. Patients in the Cohort

This was a single-center study that was performed using remnant samples. The protocol was approved by the institutional review board of Incheon St. Mary's Hospital (OC18TESI0121). We enrolled 80 consecutive sepsis patients (≥18 years of age) who were admitted to the intensive care unit (ICU) through the emergency department (ED). Eighty healthy substitutes who participated in a routine health check-up program were enrolled from August 2018 to February 2019. Patients were excluded if they had evidence of an immune compromised state (e.g., malignancy and chemotherapy administration). Demographic data, baseline characteristics, and initial laboratory data of patients were collected at the time of ICU admission through the ED (Table 1).

### 2.2. Diagnosis of Sepsis

Sepsis was diagnosed based on SEPSIS-3 criteria, which were based on SOFA (sequential organ failure score) score ≥ 2 from baseline with evidence of infection [1, 15]. The SOFA score was composed of respiratory factor, blood pressure, consciousness using the Glasgow Coma Scale, and three laboratory data (platelet count, bilirubin level, and creatinine level). Increased SOFA score correlated with severity of sepsis. Documentation of infection was defined by medical examinations as follows: microbiological tests, including culture of body fluids; real-time or conventional polymerase chain reaction; radiological analyses, including X-ray, ultrasonography, and computed tomography; and serology [12, 13].

### 2.3. Laboratory Examinations

After entering the ED, routine microbiology examination for patients included more than one pair of blood cultures. Blood samples were drawn immediately after admission to the ED before treatment and were analyzed in a central laboratory within 2 hours [13]. Analysis of various body fluids (urine, sputum, bronchoalveolar lavage fluid, cerebrospinal fluid, abscess, and closed wound) was performed according to patient status. Hematologic parameters were measured using the XN-2000 series (Sysmex Corporation, Kobe, Japan), and blood chemistry data were measured using an AU5800 Automated Biochemistry Analyzer (Beckman Coulter, Miami, FL, USA). Arterial blood gas analysis was measured using the GEM premier 3500 analyzer (Instrumentation Laboratory, Bedford, MA, USA). These laboratory data were collected during normal clinical practice.

### 2.4. Cytokine Measurements

Leftover serum samples after routine laboratory tests were collected and stored at -80°C before analysis of cytokines. Cytokines were measured simultaneously using the Cytokine/Chemokine/Growth Factor 45-plex Human ProcartaPlex Panel 1 (Thermo Fisher Scientific, Waltham, MA, USA). In brief, serum samples were thawed on ice and centrifuged at 10,000 g for 10 minutes. The addition of 50 *μ*L of beads to each plate was followed by 25 *μ*L of standards, controls, and samples. Incubation was performed for 2 hours, followed by two washes. Detection antibody (25 *μ*L) was added, and plates were incubated for 30 minutes at room temperature. Streptavidin with attached phycoerythrin was added for 30 minutes. Data was acquired using a MAGPIX instrument to measure signal intensities (Luminex Corporation, Austin, TX, USA).

Molecules included for analysis included IFN-*α*, IFN-*γ*, IL-1*β*, IL-1*α*, IL-1RA, IL-2, IL-4, IL-5, IL-6, IL-7, IL-8, IL-9, IL-10, IL-12, IL-13, IL-15, IL-17*α*, IL-18, IL-21, IL-22, IL-23, IL-27, IL-31, LIF, TNF-*α*, TNF-*β*, CCL2, CCL3, CCL4, CCL5, CCL11, CXCL1, CXCL10, CXCL12, BDNF, EGF, FGF-2, GM-CSF, HGF, NGF-*β*, PDGF-BB, PLGF-1, SCF, VEGF-A, and VEGF-D.

### 2.5. Statistical Analyses

The comparison of 80 sepsis patients and 80 normal control patients were performed using the Mann-Whitney *U* test for cytokine, chemokine, and growth factor levels. Bonferroni correction was performed for *P* value calculations. Diagnostic performance was analyzed using receiver operation characteristic (ROC) curves, which were compared using a nonparametric method. The maximum area under the ROC curve (AUC) was used as cut-off values. Sensitivity, specificity, positive and negative predictive values, and accuracies were calculated with a 95% confidence interval. Correlation analysis by Spearman's method was used to analyze cytokine related with SOFA and Acute Physiology and Chronic Health Evaluation (APACHE) score. Prognosis was predicted by logistic regression analysis. Each molecule was analyzed by the stepwise forward method, and those with statistical significance were further analyzed by multivariate analysis. Hierarchical clustering was performed using the R program to divide the data into homogenous subgroups and enlarge the difference between the subgroups [17]. The partitioned data within the same cluster were more similar to each other than to data in other clusters. Pairwise dissimilarities were calculated among samples and formed clusters that were least dissimilar between samples by calculating distances iteratively. Distance between samples were calculated by the Euclidean method [18]. Dendrogram of clustering analysis was plotted. All remaining statistical analyses were performed using MedCalc software version 18.11 (MedCalc Software bvba, Mariakerke, Belgium).

## 3. Results

### 3.1. Baseline Characteristics of Sepsis Patients

The median age (range) of the control group was 70 years (38-85) and for the sepsis group 74.5 years (38-87). In the control group, 64 individuals (80%) were >65 years of age and 16 (20%) were <65 years of age. The control group comprised 38 (47.5%) females and 42 (52.5%) males. The sepsis group comprised 36 (45%) females and 44 (55%) males (Table 1). Among sepsis patients, 53 (66.2%) had infection confirmed by bacterial growth and 27 (33.8%) had evidence of suspected bacterial infection. Among the 53 patients, bacteria were recovered from the primary site in 50, from blood culture in three, and from both primary site and blood culture in 15 (Supplemental [Supplementary-material supplementary-material-1])). Among the identified microbes that were isolated, *Escherichia coli* (12/50, 24%) was the most common pathogen, followed by *Acinetobacter baumannii* (8/50, 16%) and *Klebsiella pneumoniae* (7/50, 14%) from the primary site. In the case of blood culture, *E. coli* (9/18, 50%) was the most common pathogen, followed by the *Klebsiella* species (4/18, 22.2%). The final diagnosis revealed that 45% and 40% of all patients suffered acute respiratory tract and urinary tract infection.

### 3.2. Cytokine Profiles

Twenty-eight cytokines, eight chemokines, and nine growth factors were measured in the sepsis and control groups (Table 2). In the control group, IFN-*α*, IL-4, IL-5, IL-9, GM-CSF, and TNF-*β* were not detected. Except for EGF, concentrations of the molecules were increased in the sepsis group. Comparison of molecules between the control and sepsis groups revealed statistically significant differences, except for IFN-*α*, IL-1RA, IL-1*β*, IL-2, IL-6, IL-7, IL-15, LIF, GM-CSF, TNF-*α*, CCL4, CCL5, and CXCL12. In the control group, the levels of IL-2, IL-15, IL-27, BDNF, PDGF-BB, and PLGF-1 cytokines were significantly lower in older patients (>65, *n* = 64) compared to younger patients (≤65, *n* = 16). In the sepsis group, none of the molecules revealed statistical significance between the older and younger age group (Supplemental [Supplementary-material supplementary-material-1]).

### 3.3. Diagnosis of Sepsis

**Figure 1:**
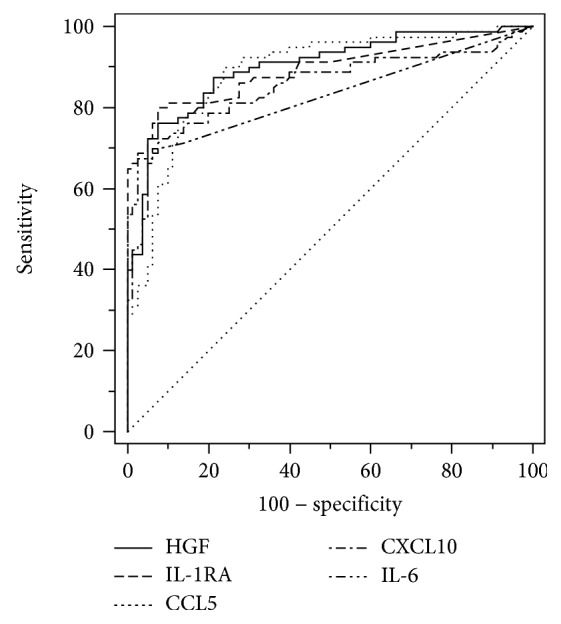
Receiver operating characteristic curves for the diagnosis of sepsis that showed the highest values among tested molecules. Comparison of HGF and IL6 (*P* = 0.042) and IL-1RA and IL-6 (*P* = 0.028) revealed statistical significance.

ROC curve analysis results for cytokines are presented in Table 3 and results for chemokine and growth factors in Table 4. HGF showed the highest area under the receiver operating characteristic curve (AUROC) value of 0.899 followed by IL-1RA (0.893), CCL5 (0.887), CXCL10 (0.851), and IL-6 (0.830). HGF and IL-1RA had higher AUROC compared to that of IL-6 by pairwise statistical comparison (Figure 1).

### 3.4. Correlation Analysis

Correlation between SOFA and APACHE score and molecules was studied using Spearman's method. Correlation coefficient and *P* value between SOFA and molecules are as follows: IL-4, 0.235 and 0.035; IL-13, 0.300 and 0.010; IL-15, -0.258 and 0.027; and SCF, 0.266 and 0.023, respectively. The respective values for the APACHE score are as follows: IL-9, 0.341 and 0.010; IL-10, -0.282 and 0.034; IL-21, 0.297 and 0.025; and NGF, 0.286 and 0.031.

### 3.5. Prediction of Nonsurvivors

**Figure 2:**
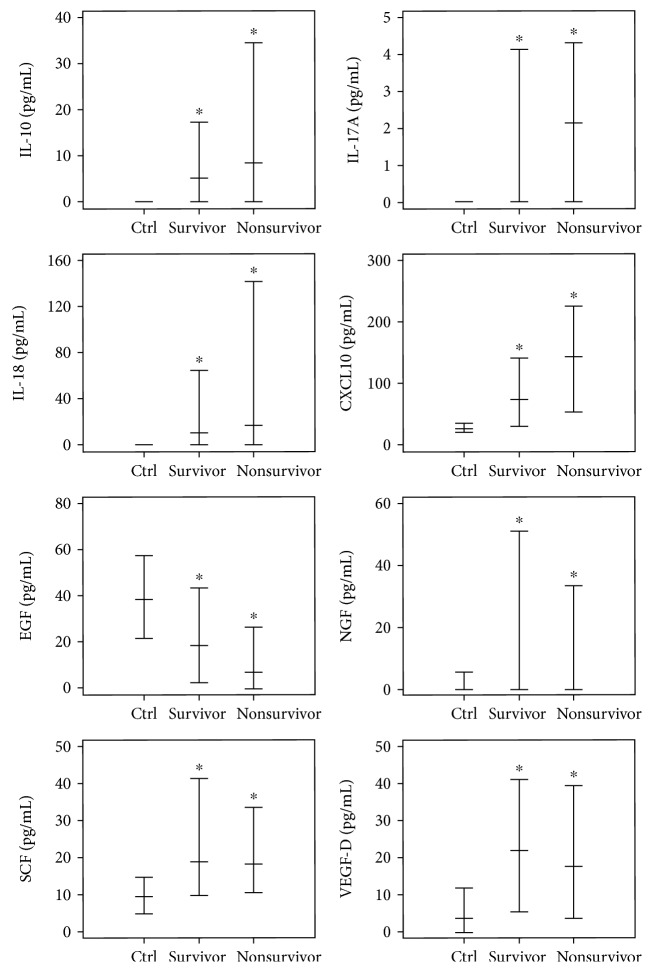
Molecular levels among the control group and survivors/nonsurvivors of sepsis. Asterisks indicate statistical significance compared to that of the control group.

Prediction of the 28-day all-cause mortality was studied by univariate and multivariate analyses. Age, SOFA score, IL-10, IL-17A, IL-18, CXCL10, EGF, NGF, SCF, and VEGF-D were included in the univariate analysis. Age, SOFA score, and EGF revealed statistical significance in multivariate analysis (Table 5). Age and SOFA score revealed an odds ratio (OR) >1.0, indicating that higher age and SOFA score predicted unfavorable prognosis. EGF revealed an OR (95% CI) of 0.979 (0.959-1.001) with a *P* value of 0.050, indicating that higher EGF levels were associated with favorable prognosis. Molecule levels revealed statistical significance when compared to the control levels. None of the molecules were significantly different in survivors versus nonsurvivors. Concentrations of EGF, NGF, SCF, and VEGF-D were decreased in nonsurvivors, whereas IL-10, IL-17A, IL-18, and CXCL10 concentrations were the same or the levels were increased in nonsurvivors (Figure 2).

### 3.6. Clustering Analysis

Supplemental [Supplementary-material supplementary-material-1] shows a hierarchical clustering analysis dendrogram. Three clusters from 80 control and 80 sepsis patient samples comprised cluster 1 (*n* = 124), cluster 2 (*n* = 5), and cluster 3 (*n* = 31). Cluster 1 revealed the lowest cytokine levels compared to those of the other groups, because all control samples were included in this cluster. Clusters 2 and 3 were composed of only sepsis patients. Cluster 2 had the highest levels of IL-1RA, IL-1*β*, IL-6, IL-7, IL-8, IL-9, IL-12, IL-13, IL-17A, Il-21, SCF, TNF-*β*, CCL4, CXCL10, CXCL12, BDNF, FGF-2, HGF, NGF, PDGF-BB, VEGF-A, and VEGF-D. Cluster 3 had the highest levels of IFN-*α*, IFN-*γ*, IL-1*α*, IL-4, IL-5, Il-10, IL-15, IL-18, Il-27, LIF, GM-CSF, TNF-*α*, CCL3, CCL5, CCL11, CXCL12, and EGF levels (Supplemental [Supplementary-material supplementary-material-1]). Cluster 1 had the lowest nonsurvivor ratio (7/115, 5.7%), cluster 2 had 40% of the nonsurvivors (2/5), and cluster 3 had 25.8% of the nonsurvivors (8/31, 25.8%). The differences were statistically significant.

## 4. Discussion

This study evaluated 45 molecules, including cytokines, chemokines, and growth factors by the multiplexing method. The focus of the definition of sepsis was changed from inflammation to organ dysfunction and immune dysregulation [1, 19]. Therefore, new biomarkers related to this definition might be required for diagnosis and prediction of prognosis. Diagnosis of sepsis under SEPSIS-3 was based on the SOFA score that includes three clinical parameters and three laboratory parameters. Diagnosis of sepsis has become sophisticated, and biomarkers that reflect infection, organ dysfunction, and immune dysregulation are required. The presence of biomarkers related with the diagnosis or prognosis of sepsis could support clinicians in the field and might enhance patient care and probability of survival. Therefore, in this study, cytokines, chemokines, and growth factors that were expected to be associated with organ dysfunction or immune dysregulation were examined.

As expected, all of the cytokines were increased in the sepsis group compared to the healthy control group, except for EGF, which was lower in the sepsis group (Table 2). In a previous study, EGF concentration was higher in those over 65 years of age in the normal healthy population [20]. Presently, however, there was no statistical significance between age >65 and <65. An age-dependent concentration difference was evident for IL-2, IL-15, IL-27, BDNF, and PDGF-BB. In the sepsis group, no molecule showed an age-dependent concentration difference.

Previous studies compared systemic inflammatory response syndrome and sepsis based on the previous SEPSIS-1 definition [16, 19]. While direct comparison of markers between the present and prior studies might be misleading, the ROC value of IL-6 was 0.830 in the present study and slightly lower (0.811) previously [12]. Unexpectedly, HGF revealed the highest ROC value of 0.899 followed by IL-1RA (0.893). In a previous study, HGF was higher in pneumonia patients compared to control, which was associated with a regenerative effect [21]. In a mouse experimental model, injected HGF blocked the apoptosis of hepatocytes against endotoxin-induced hepatic failure, whereas hepatocytes resulted in an apoptotic state in the absence of HGF injection [22]. It is thought that secreted HGF regenerates hepatocytes or other cells from apoptosis or assists in the recovery of cell damage triggered by microbes or the host immune response.

Multivariate analysis revealed age, SOFA score, and EGF as independent and statistically significant predictors of prognosis. For EGF, we hypothesize that tissue recovery or regeneration by growth factors might be associated with prognosis of sepsis. Deterioration of tissue by microbes or immune dysregulation are thought to be recovered by growth factors, such as EGF, NGF, SCF, and VEGF, or by other proteins related with damage control. However, if recovery of tissue is insufficient with these growth factors, or if growth factors are depleted, organ failure can eventually occur. Presently, the concentrations of EGF, NGF, SCF, and VEGF were decreased in nonsurvivors compared to survivors, without statistical significance (Figure 2). In an experimental mouse model of sepsis triggered by cecal ligation and puncture, systemic administration of EGF improved intestinal integrity and decreased mortality [23, 24].

To predict SOFA and APACHE II scores from the tested molecules, correlation analysis was performed. The correlation coefficient was lower, ranging from 0.2 to 0.3. These results indicated that other factors might affect organ dysfunction or disease severity. Hierarchical clustering analysis resulted in three clusters: cluster 1 (*n* = 124), cluster 2 (*n* = 5), and cluster 3 (*n* = 31). Further studies are required to determine whether cluster 2 is a real cluster or was formed by remnants after clusters 1 and 3 had formed. The rates of nonsurvivors were 5.6% (7/124), 40% (2/5), and 25.8% (8/31) in cluster 1, cluster 2, and cluster 3, respectively. Cluster 2 included four patients with urinary tract infections and one patient with a respiratory tract infection, which was statistically significant by Chi square test (*P* = 0.05). Most of the molecules were increased in clusters 2 and 3 compared to cluster 1, indicating that cytokines are increased in sepsis compared to healthy controls. Among them, EGF was lowest in cluster 2 (which had the highest 28-day mortality), lower in cluster 3, and highest in cluster 1. As EGF was the only molecule that was included in the multivariate analysis, EGF reflects prognosis and might play an important role in the pathogenesis of sepsis. IL-1*α* and IL-4 were lowest in cluster 2, which might be related with unfavorable prognosis or immune dysregulation. In a previous network analysis, the IL-4 gene was the hub node among the sepsis group, implying that this gene was related with other cytokine molecules. Although patient survival was not reported in the previous report, IL-4 is one of the molecules that plays an important role. The decreased level of IL-4 in cluster 2 might have resulted in higher mortality.

The limitations of this study are the relatively small sample sizes and the lack of proper hierarchical clustering analysis. Age and sex between the control and sepsis groups were not perfectly matched and the age of the patient group was slightly higher than that of the control group; age is a known risk factor for sepsis. As there are scant data related to growth factors in prior studies, further studies will be required to verify the present results.

## 5. Conclusions

HGF and IL-1RA demonstrated diagnostic capability, and EGF predicted favorable prognosis among sepsis patients. Most of the growth factors were decreased in nonsurvivors of sepsis, which may be associated with the pathogenesis of sepsis. Further studies are required to verify the use of cytokines, chemokines, and growth factors for sepsis diagnosis and prediction of prognosis.

## Figures and Tables

**Table 1:** Clinical characteristics and baseline demographics of the 80 sepsis patients^a^.

Characteristics		
*Demographics*		
Female/male (*n*, %)	36/44	(45/55%)
Age (years, range)	74.5	(38-87)
Age >65/<65	58/22	(72.5/27.5%)
*Clinical parameters*		
APACHE II score	26.2	(3-38.9)
SOFA score	4	(2-13)
*Laboratory data*		
PaO_2_/FiO_2_	140	(45-920)
Lactate (mmol/L)	2.1	(0.3-14.4)
White blood cells (×10^9^/L)	12.8	(0.42-51.55)
Platelet (×10^9^/L)	196	(9-445)
Hemoglobin (g/dL)	11.4	(4.3-17.3)
Hematocrit (%)	33.8	(12.5-49.2)
Prothrombin time in INR, ratio	1.18	(0.93-3.62)
Total bilirubin (mg/dL)	0.8	(0.3-3.8)
AST (U/dL)	27	(8-1470)
ALT (U/dL)	19	(5-2100)
Lactate dehydrogenase (IU/dL)	521	(138-2492)
BUN (mg/dL)	24.05	(8.2-184.1)
Creatinine (mg/dL)	1.06	(0.3-23.8)
*Final diagnosis*		
Urinary tract infection (*n*, %)	32	(40%)
Respiratory tract infection (*n*, %)	36	(45%)
Digestive tract infection (*n*, %)	4	(5%)
Hepatobiliary tract infection (*n*, %)	3	(3.7%)
Others (*n*, %)	5	(6.3%)
*Underlying disease*		
HTN/DM (*n*, %)	10/6	(12.5/7.5%)
HTN + DM (*n*, %)	13	(16.3%)
*Prognosis*		
Survivor (*n*, %)	63	(78.7%)
Nonsurvivor (*n*, %)	17	(21.2%)

^a^Data are presented as median (range) for continuous variables and frequency (%) for categorical variables. APACHE: acute physiology and chronic health evaluation; SOFA: sequential organ failure assessment; AST: aspartate aminotransferase; ALT: alanine aminotransferase; HTN: hypertension; DM: diabetes mellitus.

**Table 2:** Comparison of cytokines, chemokines, and growth factors between control and sepsis groups.

Molecules (pg/mL)	Control (*n* = 80)		Sepsis (*n* = 80)		Bonferroni
	Mean	SD	Mean	SD	correction (*P* value)
Cytokines					
IFN-*α*	0.05	0.48	7.82	63.18	NS
IFN-*γ*	0.00	0.00	20.82	142.23	<0.05
IL-1RA	216.65	348.15	17564.94	32132.31	NS
IL-1*α*	2.18	7.23	18.02	55.50	<0.05
IL-1*β*	0.45	1.62	3.26	7.92	NS
IL-2	10.57	10.26	17.40	24.54	NS
IL-4	0.00	0.00	8.88	33.03	<0.05
IL-5	0.00	0.00	4.95	18.71	<0.05
IL-6	4.72	17.12	1931.93	4803.73	NS
IL-7	2.21	1.94	6.42	7.94	NS
IL-8	12.43	36.10	310.08	945.84	<0.05
IL-9	0.00	0.00	13.63	96.09	<0.05
IL-10	0.07	0.44	88.55	321.44	<0.05
IL-12p70	0.19	1.72	0.36	2.39	<0.05
IL-13	0.48	1.94	6.77	19.59	<0.05
IL-15	10.35	12.49	17.45	31.95	NS
IL-17A	1.04	2.44	7.05	16.59	<0.05
IL-18	1.50	5.51	53.49	85.48	<0.05
IL-21	5.12	19.14	102.14	460.64	<0.05
IL-22	10.06	45.38	494.81	2857.13	<0.05
IL-23	2.11	15.13	58.33	272.71	<0.05
IL-27	0.99	8.77	46.00	201.57	<0.05
IL-31	0.65	5.75	30.31	158.07	<0.05
LIF	6.22	7.71	45.19	131.78	NS
GM-CSF (CSF2)	0.00	0.00	6.71	40.87	NS
SCF (KITLG)	11.12	8.92	28.70	34.10	<0.05
TNF-*α* (LTA)	1.64	5.73	4.43	14.96	NS
TNF-*β* (LTB)	0.00	0.00	9.48	72.78	<0.05
Chemokines					
CCL2 (MCP-1)	65.96	48.52	811.06	1335.39	<0.05
CCL3 (MIP-1*α*)	16.07	21.33	22.90	36.19	<0.05
CCL4 (MIP-1*β*)	264.96	163.90	729.02	1870.12	NS
CCL5 (RANTES)	185.57	185.20	604.49	565.59	NS
CCL11 (eotaxin)	112.30	49.84	126.04	100.40	<0.05
CXCL1 (GRO-*α*)	1.81	11.51	82.01	301.55	<0.05
CXCL10 (IP-10)	31.98	18.56	234.04	549.42	<0.05
CXCL12 (SDF-1)	691.12	207.49	1325.08	1204.70	NS
Growth factors					
BDNF	262.82	226.55	604.23	589.93	<0.05
EGF	43.62	30.99	35.90	58.03	<0.05
FGF-2	0.08	0.74	28.01	110.35	<0.05
HGF	184.27	142.38	1111.45	1570.22	<0.05
NGF	7.46	15.35	31.18	50.72	<0.05
PDGF-BB	119.25	132.24	565.48	1157.25	<0.05
PLGF-1	141.03	86.31	237.79	271.32	<0.05
VEGF-A	204.48	212.45	831.87	1088.99	<0.05
VEGF-D (FIGF)	6.84	8.46	27.58	28.21	<0.05

IFN: interferon; IL: interleukin; LIF: leukemia inhibitory factor; TNF: tumor necrosis factor; CCL: CC motif chemokine ligand; CXCL: CXC motif ligand; BDNF: brain-derived neurotrophic factor; EGF: epidermal growth factor; FGF: fibroblast growth factor; GM-CSF: granulocyte macrophage colony stimulating factor; HGF: hepatocyte growth factor; NGF: nerve growth factor; PDGF: platelet-derived growth factor; PLGF: placental growth factor; SCF: stem cell factor; VEGF: vascular endothelial growth factor.

**Table 3:** Receiver operating characteristic curve analysis of 28 cytokines for diagnosis of sepsis.

Biomarkers	AUC	Cut-off	Sensitivity	(95% CI)	Specificity	(95% CI)	PPV	(95% CI)	NPV	(95% CI)	Accuracy	(95% CI)
IFN-*α*	0.531	1.0	7.5	(5.8-9.1)	98.8	(98.1-99.4)	86.2	(40.5-62.7)	51.6	(40.5-62.7)	53.2	(44.7-61.5)
IFN-*γ*	0.556	1.0	11.3	(9.3-13.2)	100	(99.9-100)	100	(99.9-100)	0.10	(3.4-16.8)	19.3	(12.6-26.1)
IL-1RA	0.893	581.6	80.1	(77.6-82.5)	92.5	(90.8-94.1)	91.4	(84.2-98.5)	82.2	(73.8-90.7)	86.3	(80.5-92.1)
IL-1*β*	0.598	1.9	27.5	(24.7-30.2)	96.3	(95.1-97.4)	88.1	(79.8-96.3)	43.1	(46.1-68.1)	61.9	(53.7-70.1)
IL-1*α*	0.672	1.4	47.4	(44.3-50.4)	87.5	(85.4-89.5)	79.1	(68.7-89.5)	62.4	(51.7-73.2)	67.4	(59.5-75.3)
IL-2	0.583	1.0	75.0	(72.3-77.6)	38.8	(35.8-41.8)	55.1	(42.3-67.7)	60.8	(49.9-71.6)	56.9	(48.5-62.5)
IL-4	0.556	1.0	11.3	(9.3-13.2)	100	(99.9-100)	100	(99.9-100)	52.9	(41.9-64.1)	55.6	(47.2-64.1)
IL-5	0.575	1.0	15.1	(12.8-17.3)	100	(99.9-100)	100	(99.9-100)	54.1	(43.0-65.1)	57.6	(59.2-65.9)
IL-6	0.830	40.8	67.5	(64.5-70.4)	95.5	(94.2-96.8)	93.7	(87.6-99.9)	74.6	(64.9-84.2)	81.5	(74.9-88.1)
IL-7	0.707	3.2	57.5	(54.4-60.5)	85.1	(82.8-87.3)	79.4	(69.1-89.7)	66.7	(56.2-77.1)	71.3	(63.7-78.9)
IL-8	0.781	11.2	71.3	(68.4-74.1)	81.3	(78.8-83.7)	79.2	(68.8-89.6)	73.9	(64.1-83.4)	76.3	(69.1-83.4)
IL-9	0.519	1.0	3.8	(2.6-4.9)	100	(99.9-100)	100	(99.9-100)	50.9	(39.8-62.1)	51.9	(43.4-60.3)
IL-10	0.820	1.0	65.1	(62.1-68.1)	97.5	(96.5-98.5)	96.3	(91.4-100)	73.6	(63.8-83.4)	81.3	(74.7-87.9)
IL-12	0.506	1.0	2.5	(1.5-3.5)	98.7	(97.9-99.4)	65.7	(53.6-77.8)	50.3	(39.2-61.4)	50.6	(42.2-59.1)
IL-13	0.606	10.5	21.3	(18.7-23.8)	100	(99.9-100)	100	(99.9-100)	55.9	(44.9-66.9)	60.6	(52.4-68.8)
IL-15	0.513	24.7	25.0	(22.1-27.3)	91.3	(89.5-93.0)	73.9	(62.7-85.1)	54.8	(43.7-65.8)	58.0	(49.6-66.3)
IL-17A	0.641	2.15	42.5	(39.4-45.6)	88.8	(86.8-90.7)	79.1	(68.7-89.5)	60.7	(49.8-71.5)	65.7	(57.6-73.6)
IL-18	0.746	1.0	53.8	(50.7-56.8)	92.5	(90.8-94.1)	87.7	(79.4-96.1)	66.7	(56.2-77.1)	73.1	(65.6-80.6)
IL-21	0.685	1.0	23.8	(21.1-26.4)	92.5	(90.8-94.1)	76.0	(65.1-86.9)	54.8	(43.7-65.8)	58.1	(49.8-66.4)
IL-22	0.615	1.0	28.7	(25.8-31.5)	93.8	(92.3-95.2)	82.2	(72.4-91.9)	56.8	(45.8-67.8)	61.2	(53.0-69.4)
IL-23	0.601	1.0	22.5	(19.9-25.1)	97.5	(96.5-98.4)	90.0	(82.3-97.6)	55.7	(44.6-66.7)	60.0	(51.7-68.2)
IL-27	0.613	1.0	23.8	(21.1-26.4)	98.7	(97.9-99.4)	94.8	(89.1-100)	56.4	(45.4-67.4)	61.2	(53.0-69.4)
IL-31	0.575	1.0	16.5	(14.1-18.8)	98.8	(98.1-99.4)	93.2	(86.8-99.6)	54.2	(43.1-65.2)	57.6	(49.3-65.9)
LIF	0.760	6.1	78.8	(76.2-81.3)	62.5	(59.4-65.5)	67.7	(55.8-79.6)	74.7	(65.0-84.2)	70.6	(62.9-78.3)
GM-CSF (CSF2)	0.519	1.0	3.8	(2.6-4.9)	100	(99.0-100)	100	(99.0-100)	50.9	(39.8-62.0)	51.9	(43.5-60.3)
SCF (KITLG)	0.721	14.8	60.1	(57.1-63.1)	77.5	(74.9-80.1)	72.7	(61.4-84.1)	66.0	(55.5-76.5)	68.8	(60.9-76.6)
TNF-*α* (LTA)	0.531	1.0	15.1	(12.8-17.3)	91.3	(89.5-93.0)	63.4	(51.2-75.7)	51.8	(40.7-62.9)	53.2	(44.7-61.6)
TNF-*β* (LTB)	0.513	1.0	2.5	(1.5-3.4)	100	(99.0-100)	100	(99.0-100)	50.6	(39.5-61.7)	51.3	(42.8-59.6)

AUC: area under receiver operating characteristic curve; PPV: positive predictive value; NPV: negative predictive value: CI; confidence intervals; IFN: interferon; IL: interleukin; LIF: leukemia inhibitory factor; TNF: tumor necrosis factor; GM-CSF: granulocyte macrophage colony stimulating factor; SCF: stem cell factor.

**Table 4:** Receiver operating characteristic curve analysis of eight chemokines and nine growth factors for diagnosis of sepsis.

Biomarkers	AUC	Cut-off	Sensitivity	(95% CI)	Specificity	(95% CI)	PPV	(95% CI)	NPV	(95% CI)	Accuracy	(95% CI)
Chemokines												
CCL2 (MCP-1)	0.840	105.5	67.5	(64.5-70.4)	93.0	(91.5-94.5)	0.90	(82.3-97.6)	75.4	(65.9-85.1)	80.7	(74.1-87.3)
CCL3 (MIP-1*α*)	0.530	9.8	52.5	(49.4-55.6)	56.3	(53.2-59.3)	54.5	(41.8-67.3)	54.2	(41.8-67.2)	75.4	(45.9-62.8)
CCL4 (MIP-1*β*)	0.653	411.8	47.5	(44.3-50.5)	87.5	(85.5-89.5)	79.2	(68.8-89.5)	62.5	(51.7-73.2)	67.5	(59.9-75.4)
CCL5 (RANTES)	0.887	179.6	90.1	(89.2-92.7)	76.3	(64.3-70.2)	73.3	(62.0-84.6)	88.3	(81.2-95.4)	79.1	(72.2-85.9)
CCL11 (eotaxin)	0.508	49.6	22.5	(19.9-25.1)	93.7	(92.1-95.2)	78.1	(67.5-88.6)	54.7	(43.6-65.7)	58.1	(49.8-66.4)
CXCL1 (GRO-*α*)	0.759	1.0	55.0	(51.9-58.1)	96.3	(95.1-97.4)	93.6	(87.4-99.8)	68.1	(57.8-78.5)	75.6	(68.4-82.8)
CXCL10 (IP-10)	0.851	46.5	71.3	(68.4-74.1)	92.5	(90.8-94.1)	90.4	(82.9-97.8)	76.3	(66.8-85.8)	81.9	(75.4-88.4)
CXCL12 (SDF-1)	0.766	895.6	60.1	(57.1-63.1)	90.1	(88.2-91.9)	85.9	(76.9-94.7)	69.3	(59.0-79.5)	75.1	(67.8-82.3)
Growth factors												
BDNF	0.690	393.3	57.5	(54.4-60.5)	82.5	(80.1-84.8)	76.6	(65.8-87.4)	66.0	(55.4-76.5)	70.0	(62.2-77.7)
EGF	0.663	20.4	58.8	(55.7-61.8)	77.5	(74.9-80.8)	72.3	(60.9-83.7)	65.3	(54.7-75.8)	68.2	(60.3-76.0)
FGF-2	0.601	1.0	21.5	(18.9-24.0)	98.8	(98.1-99.4)	94.7	(89.1-100)	55.7	(44.7-66.7)	60.2	(51.9-68.4)
HGF	0.899	341.2	76.3	(73.6-78.9)	92.5	(90.8-94.1)	91.1	(83.7-98.3)	79.6	(70.1-88.5)	84.4	(78.2-90.5)
NGF	0.636	21.9	40.1	(37.1-43.1)	86.3	(84.2-88.4)	74.5	(63.4-85.6)	59.1	(48.1-69.9)	63.2	(55.1-71.3)
PDGF-BB	0.677	279.6	38.8	(35.7-41.8)	90.1	(88.2-91.5)	79.6	(69.4-89.9)	59.5	(48.6-70.4)	64.4	(56.3-72.5)
PLGF-1	0.626	87.2	81.3	(78.8-83.7)	41.3	(38.2-44.3)	58.1	(45.4-70.6)	68.8	(58.6-79.1)	61.3	(53.1-69.5)
VEGF-A	0.790	313.5	67.5	(64.5-70.4)	80.1	(77.6-82.5)	77.2	(66.5-87.9)	71.1	(61.1-81.1)	73.8	(66.8-81.2)
VEGF-D (FIGF)	0.772	13.8	63.5	(60.5-66.4)	83.8	(81.5-88.1)	79.6	(69.4-89.9)	69.6	(59.4-79.8)	73.7	(66.2-81.1)

AUC: area under receiver operating characteristic curve; PPV: positive predictive value; NPV: negative predictive value: CI; confidence intervals; CCL: CC motif chemokine ligand; CXCL: CXC motif ligand; BDNF: brain-derived neurotrophic factor; EGF: epidermal growth factor; FGF: fibroblast growth factor; GM-CSF: granulocyte macrophage colony stimulating factor; HGF: hepatocyte growth factor; NGF: nerve growth factor; PDGF: platelet-derived growth factor; PLGF: placental growth factor; SCF: stem cell factor; VEGF: vascular endothelial growth factor.

**Table 5:** Univariate and multivariate analysis for the prediction of prognosis among sepsis patients.

	Univariate				Multivariate			
	Odds ratio	95% CI		*P* value	Odds ratio	95% CI		*P* value
Age	1.058	0.999	1.121	0.038	1.092	1.012	1.177	0.022
AST				NS				
ALT				NS				
CRP				NS				
PCT				NS				
Creatinine				NS				
Total bilirubin				NS				
APACHE II score				NS				
SOFA score	1.279	1.064	1.539	0.009	1.349	1.074	1.695	0.010
IL-10	1.001	1.000	1.002	0.034				
IL-17A	1.019	1.001	1.037	0.036				
IL-18	1.010	1.000	1.019	0.047				
CXCL10	1.002	1.000	1.004	0.014				
EGF	0.973	0.957	0.989	0.001	0.979	0.959	1.001	0.050
NGF	0.984	0.974	0.995	0.005				
SCF	0.975	0.958	0.993	0.007				
VEGF-D	0.998	0.975	1.000	0.050				

AST: aspartate aminotransferase; ALT: alanine aminotransferase; CRP: C-reactive protein; PCT: procalcitonin; APACHE: acute physiology and chronic health evaluation; SOFA: sequential organ failure assessment; IL: interleukin; CXCL: chemokine (C-X-C motif) ligand; EGF: epidermal growth factor; NGF: nerve growth factor; SCF: stem cell factor; VEGF-D: vascular endothelial growth factor D precursor.

## Data Availability

The data used to support the findings of this study are included within the supplementary information files.

## Supplementary Materials

Supplementary Materials Supplementary Figure 1: dendrogram of clustering analysis that resulted in 3 clusters. Supplementary Table 1: cultured pathogens from sepsis patients. Supplementary Table 2: cytokine levels in the normal control group that showed statistical significance tested by the Mann-Whitney *U* test. Supplementary Table 3: cytokine concentrations among clusters and comparison of clusters.

## References

[1] Shankar-Hari M., Phillips G. S., Levy M. L. (2016). Developing a new definition and assessing new clinical criteria for septic shock: for the Third International Consensus Definitions for Sepsis and Septic Shock (Sepsis-3). *JAMA*.

[2] Gotts J. E., Matthay M. A. (2016). Sepsis: pathophysiology and clinical management. *BMJ*.

[3] Fleischmann C., Scherag A., Adhikari N. K. J. (2016). Assessment of global incidence and mortality of hospital-treated sepsis. Current estimates and limitations. *American Journal of Respiratory and Critical Care Medicine*.

[4] Delano M. J., Ward P. A. (2016). Sepsis-induced immune dysfunction: can immune therapies reduce mortality?. *The Journal of Clinical Investigation*.

[5] Schulte W., Bernhagen J., Bucala R. (2013). Cytokines in sepsis: potent immunoregulators and potential therapeutic targets—an updated view. *Mediators of Inflammation*.

[6] Angus D. C., Opal S. (2016). Immunosuppression and secondary infection in sepsis: part, not all, of the story. *JAMA*.

[7] Dellinger R. P., Levy M. M., Rhodes A. (2013). Surviving Sepsis Campaign: international guidelines for management of severe sepsis and septic shock, 2012. *Intensive Care Medicine*.

[8] Delano M. J., Ward P. A. (2016). The immune system’s role in sepsis progression, resolution, and long-term outcome. *Immunological Reviews*.

[9] Larsen F. F., Petersen J. A. (2017). Novel biomarkers for sepsis: a narrative review. *European Journal of Internal Medicine*.

[10] van der Poll T., van de Veerdonk F. L., Scicluna B. P., Netea M. G. (2017). The immunopathology of sepsis and potential therapeutic targets. *Nature Reviews. Immunology*.

[11] Vermont C. L., Hazelzet J. A., de Kleijn E. D., de Groot R. (2006). CC and CXC chemokine levels in children with meningococcal sepsis accurately predict mortality and disease severity. *Critical Care*.

[12] Jekarl D. W., Kim J. Y., Lee S. (2015). Diagnosis and evaluation of severity of sepsis via the use of biomarkers and profiles of 13 cytokines: a multiplex analysis. *Clinical Chemistry and Laboratory Medicine (CCLM)*.

[13] Jekarl D. W., Lee S. Y., Lee J. (2013). Procalcitonin as a diagnostic marker and IL-6 as a prognostic marker for sepsis. *Diagnostic Microbiology and Infectious Disease*.

[14] Hotchkiss R. S., Monneret G., Payen D. (2013). Sepsis-induced immunosuppression: from cellular dysfunctions to immunotherapy. *Nature Reviews Immunology*.

[15] Seymour C. W., Liu V. X., Iwashyna T. J. (2016). Assessment of clinical criteria for sepsis: for the Third International Consensus Definitions for Sepsis and Septic Shock (Sepsis-3). *JAMA*.

[16] Bone R. C., Balk R. A., Cerra F. B. (1992). Definitions for sepsis and organ failure and guidelines for the use of innovative therapies in sepsis. *Chest*.

[17] Hastie T., Tibshirani R., Friedman J. (2009). *The elements of statistical learning: data mining, inference and prediction*.

[18] Crawley M. J. (2013). *The R Book*.

[19] Singer M., Deutschman C. S., Seymour C. W. (2016). The Third International Consensus Definitions for Sepsis and Septic Shock (Sepsis-3). *JAMA*.

[20] Kim H. O., Kim H. S., Youn J. C., Shin E. C., Park S. (2011). Serum cytokine profiles in healthy young and elderly population assessed using multiplexed bead-based immunoassays. *Journal of Translational Medicine*.

[21] Nayeri F., Darelid J., Nilsson I. (2002). Hepatocyte growth factor may act as an early therapeutic predictor in pneumonia. *Scandinavian Journal of Infectious Diseases*.

[22] Kosai K., Matsumoto K., Funakoshi H., Nakamura T. (1999). Hepatocyte growth factor prevents endotoxin-induced lethal hepatic failure in mice. *Hepatology*.

[23] Clark J. A., Clark A. T., Hotchkiss R. S., Buchman T. G., Coopersmith C. M. (2008). Epidermal growth factor treatment decreases mortality and is associated with improved gut integrity in sepsis. *Shock*.

[24] Marshall J. C. (2008). Sepsis: rethinking the approach to clinical research. *Journal of Leukocyte Biology*.

