# Supplementary material for: Diagnosis and Prognosis of Sepsis Based on Use of Cytokines, Chemokines, and Growth Factors
Source: Dis Markers. 2019 Sep 8;2019:1089107. doi: 10.1155/2019/1089107 (PMC6754872; doi:10.1155/2019/1089107)

Supplemental Table 1. Cultured pathogens from sepsis patients

Pathogen	N
Gram positive	
Staphylococcus aureus	6
Enterobacter spp.	4
Sreptococcus pneumoniae	1
Gram negative	
Escherichia coli	12
Klebsiella pneumoniae	9
Acinetobacter baumannii	8
Pseudomonas aeruginosa	7
Proteus mirabilis	4
Morganella morganii	1
Salmonella spp	1

Supplemental Table 2 Cytokine levels in normal control group that showed statistical significance by Mann-Whitney U test

	Age group, years	N	Mean	Standard deviation
IL-2	≤65	16	16.34	7.91
	>65	64	8.96	10.31
IL-15	≤65	16	18.00	15.06
	>65	64	8.27	11.03
IL-27	≤65	16	4.87	19.48
	>65	64	0.00	0.00
BDNF	≤65	16	142.79	291.08
	>65	64	40.80	130.23
PDGF-BB	≤65	16	79.70	137.69
	>65	64	18.87	52.10

Supplemental Table 3 Cytokine concentrations among clusters and comparison of clusters

Molecules	Cluster 1	Cluster 2	Cluster 3	1 vs 2	2 vs 3	1 vs 3
Cytokines						
IFN-α	0.03±0.38	0	50.26±241.44			<0.001
IFN-γ	6.46±55.81	0	37.47±117.59			
IL-1RA	324.59±944.30	90766.57±128336.37	2824.21±7812.53			
IL-1β	81.40±341.21	81772.34±115643.56	4589.83±13138.40	0.01		
IL-1α	111.08±595.99	6.25±1.09	10077.10±16520.51	0.006		<0.001
IL-2	10.38±235.69	32.36±10.88	11.41±37.15	0.013	0.009	
IL-4	374.64±1441.92	29.18±26.03	12069.11±24716	0.008		<0.001
IL-5	1.75±8.35	13.08±15.54	14.11±26.31	<0.001		<0.001
IL-6	10.37±33.22	195.43±283.86	163.58±659.55	<0.001		
IL-7	9.03±30.93	9466.05±10938.06	371.77±1586.27	0.043		
IL-8	55.26±409.15	6118.83±12125.17	1375.96±3992.14	0.002		<0.001
IL-9	3.64±15.91	378.41±550.49	32.67±66.94	<0.001		<0.001
IL-10	38.10±267.73	894.67±1768.19	956.24±2036.06	0.01		<0.001
IL-12p70	0.71±2.12	556.22±770.41	11.01±39.63			<0.001
IL-13	3.25±13.85	1552.05±2447.52	140.63±456.11			<0.001
IL-15	8.80±0.1	9.41±10.16	30.63±149.17			
IL-17A	3.45±9.00	32.45±29.64	20.16±34.86	0.001		<0.001
IL-18	6.04±20.65	14.96±16.10	32.14±66.15	0.01		<0.001
IL-21	11.59±45.66	89.53±117.56	23.27±39.92	0.003		<0.001
IL-22	21.1±91.42	262.31±467.33	69.48±192.62	0.001		0.002
IL-23	22.8±154.42	724.03±1348.31	274.03±974.71	<0.001		<0.001
IL-27	16.83±75.01	199.22±296.47	849.62±4510.31	<0.001		<0.001
IL-31	3.99±19.27	50.54±45.96	50.12±152.40	<0.001		<0.001
LIF	10.96±33.64	71.93±123.01	111.51±373.25			
GM-CSF (CSF2)	0.06±0.61	5.71±12.76	15.35±68.66	0.001		0.04
SCF (KITLG)	83.78±82.45	78.10±96.12	32.25±23.26			0.001
TNF-α (LTA)	2.73±7.94	46.17±50.33	96.04±416.02	0.001		
TNF-β (LTB)	0.93±5.64	199.73±433.83	14.22±23.59	<0.001		<0.001
Chemokines						
CCL2 (MCP-1)	146.62±218.59	2978.34±1701.76	766.09±1081.83	0.005		<0.001
CCL3 (MIP-1α)	92.10±317.88	120.32±54.90	242.30±427.63	0.005		<0.001
CCL4 (MIP-1β)	328.89±504.56	1743.79±1382.48	547.83±613.79	0.017		
CCL5 (RANTES)	354.51±912.42	538.14±321.84	1265.71±3628.00			
CCL11 (Eotaxin)	173.82±210.18	159.41±90.89	366.70±278.58			<0.001
CXCL1 (GRO-α)	66.30±238.37	223.36±163.92	362.02±837.42	0.001		<0.001
CXCL10 (IP-10)	58.26±104.27	409.06±493.48	180.81±253.36	0.001		<0.001
CXCL12 (SDF-1)	703.59±492.57	2296.96±1624.28	729.21±847.58	0.0023	0.03	
Growth factors						
BDNF	214.23±375.93	937.66±950.02	702.81±596.03	0.007		<0.001
EGF	153.95±185.65	44.79±34.95	49.16±78.35			<0.001
FGF-2	25.12±33.02	29.29±40.12	27.82±68.44			
HGF	251.43±432.42	2482.39±1614.65	1637.04±2343.02	<0.001		<0.001
NGF	111.03±137.94	46.49±36.48	34.76±55.73			0.001
PDGF-BB	144.04±469.85	2043.20±3406.57	769.97±960.02	0.003		<0.001
PLGF-1	143.88±149.30	535.78±757.60	300.93±277.85	0.025		
VEGF-A	239.01±574.98	1918.95±1363.06	1125.42±1466.87	0.001		<0.001
VEGF-D (FIGF)	111.09±167.33	54.17±35.37	33.96±24.76			

IFN, interferon; IL, interleukin; LIF, leukemia inhibitory factor; TNF, tumor necrosis factor; CCL, CC motif chemokine ligand; CXCL, CXC motif ligand; BDNF, brain derived neurotrophic factor; EGF, epidermal growth factor; FGF, fibroblast growth factor; GM-CSF, granulocyte macrophage colony stimulating factor; HGF, hepatocyte growth factor; NGF, nerve growth factor; PDGF, platelet derived growth factor; PLGF, placental growth factor ; SCF, stem cell factor; VEGF, vascular endothelial growth factor

Supplemental Figure 1. Dendrogram of clustering analysis that resulted in three clusters

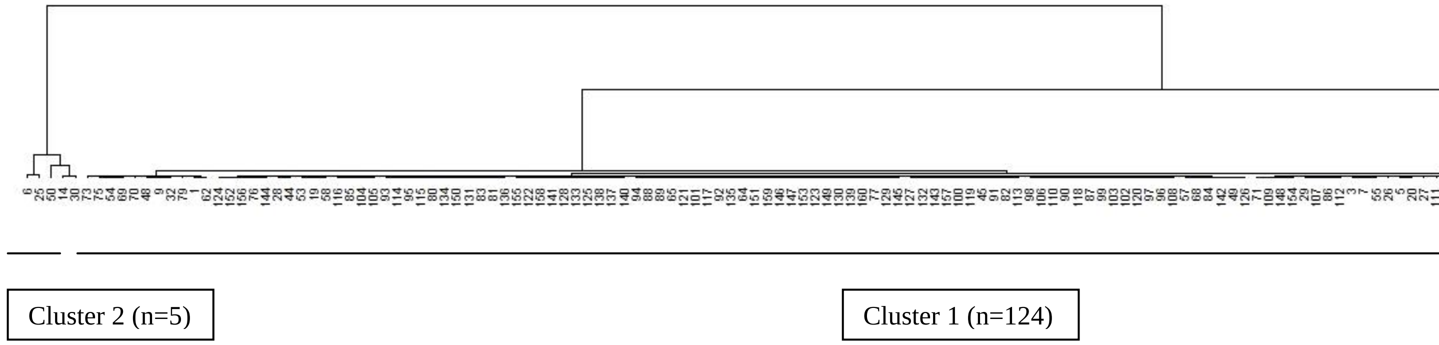

Supplement: Supplementary Materials — Supplementary Figure 1: dendrogram of clustering analysis that resulted in 3 clusters. Supplementary Table 1: cultured pathogens from sepsis patients. Supplementary Table 2: cytokine levels in the normal control group that showed statistical significance tested by the Mann-Whitney U test. Supplementary Table 3: cytokine concentrations among clusters and comparison of clusters. [file 1089107.f1.pdf]
